# Non-covalent Methods of Engineering Optical Sensors Based on Single-Walled Carbon Nanotubes

**DOI:** 10.3389/fchem.2019.00612

**Published:** 2019-09-19

**Authors:** Alice J. Gillen, Ardemis A. Boghossian

**Affiliations:** École Polytechnique Fédérale de Lausanne, Lausanne, Switzerland

**Keywords:** optical biosensing, near-infrared sensors, single-walled carbon nanotubes (SWCNTs or SWNTs), molecular recognition, selectivity, fluorescence brightness, non-covalent solubilization

## Abstract

Optical sensors based on single-walled carbon nanotubes (SWCNTs) demonstrate tradeoffs that limit their use in *in vivo* and *in vitro* environments. Sensor characteristics are primarily governed by the non-covalent wrapping used to suspend the hydrophobic SWCNTs in aqueous solutions, and we herein review the advantages and disadvantages of several of these different wrappings. Sensors based on surfactant wrappings can show enhanced quantum efficiency, high stability, scalability, and diminished selectivity. Conversely, sensors based on synthetic and bio-polymer wrappings tend to show lower quantum efficiency, stability, and scalability, while demonstrating improved selectivity. Major efforts have focused on optimizing sensors based on DNA wrappings, which have intermediate properties that can be improved through synthetic modifications. Although SWCNT sensors have, to date, been mainly engineered using empirical approaches, herein we highlight alternative techniques based on iterative screening that offer a more guided approach to tuning sensor properties. These more rational techniques can yield new combinations that incorporate the advantages of the diverse nanotube wrappings available to create high performance optical sensors.

**GRAPHICAL ABSTRACT:**
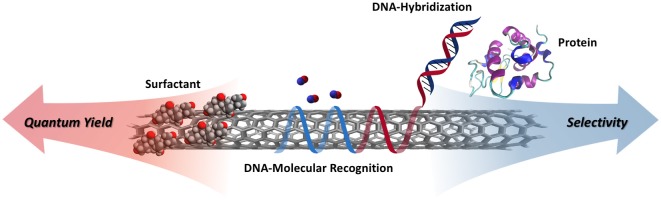
Schematic illustrating the tradeoffs between quantum yield and selectivity for various non-covalent surface functionalizations.

## 1. Introduction

Optical sensors use light as a means of contactless detection for real-time sensing. Distinct optical signals from a single device enables multimodal detection of several analytes simultaneously, a feature that is especially advantageous for remote *in vivo* biosensing applications. Fluorescence-based optical sensors require two elements for operation: a molecular recognition element that selectively interacts with the analyte of interest and an optical transducer, such as a fluorophore, that converts this interaction into a measurable optical signal.

As described in several reviews (Boghossian et al., 2011; Liu et al., 2011; Kruss et al., 2013; Pan et al., 2017), single-walled carbon nanotubes (SWCNTs) are among the most promising fluorescence-based transducers for biosensing applications. They are one-dimensional nanostructures with optoelectronic properties that are tuned by tube diameter as a result of quantum confinement. Conceptualized as cylindrically rolled sheets of graphene, SWCNTs exist with various diameters, and they can be either metallic, semi-metallic, or semiconducting, depending on the direction the sheet is rolled. In 2002, O'Connell et al. demonstrated that semiconducting forms of SWCNTs dispersed in aqueous solutions emit photoluminescence at near-infrared (near-IR) wavelengths (O'Connell et al., 2002). This emission lies within the optical transparency window for biological material (Boghossian et al., 2011) which, when coupled with the nanotube's indefinite photostability and capabilities for single-molecule detection, makes SWCNTs attractive for *in vivo* continuous monitoring applications.

**Figure 1:**
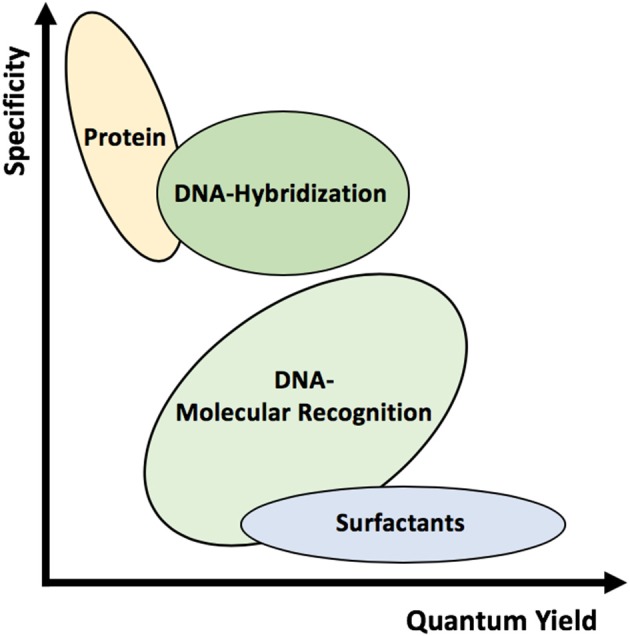
Schematic representation of the various wrappings used to suspend SWCNTs. Different wrappings can alter the quantum yield and specificity of the complexes.

The use of SWCNTs as fluorescent transducers requires surface functionalization to impart optical stability and molecular recognition. Non-functionalized SWCNTs are inherently hydrophobic and exhibit a strong tendency to aggregate into bundles in aqueous solutions. Since most SWCNT preparations contain metallic nanotubes, these bundles contribute to the fluorescence quenching of semiconducting SWCNTs through the non-radiative exciton decay channels within the bundle (O'Connell et al., 2002; Maeda et al., 2004). Therefore, the bundles need to be exfoliated to generate individually suspended nanotubes in a liquid phase for most practical applications (Coleman, 2009). Specifically, this suspension allows the semi-conducting nanotubes to fluoresce in the near-IR. In addition to enabling solubilization and fluorescence, surface functionalization can also modify the nanotube surface to promote selective interactions with particular analytes of interest (Figure 1). The underlying mechanism for this selectivity depends on the wrapping and remains an ongoing area of research for many wrappings (Jeng et al., 2006; Hertel et al., 2010; Bisker et al., 2016; Polo and Kruss, 2016; Antonucci et al., 2017; Kruss et al., 2017; Mann et al., 2017; Zubkovs et al., 2017; Gillen et al., 2018; Wu et al., 2018; Lambert et al., 2019).

**Figure 2:**
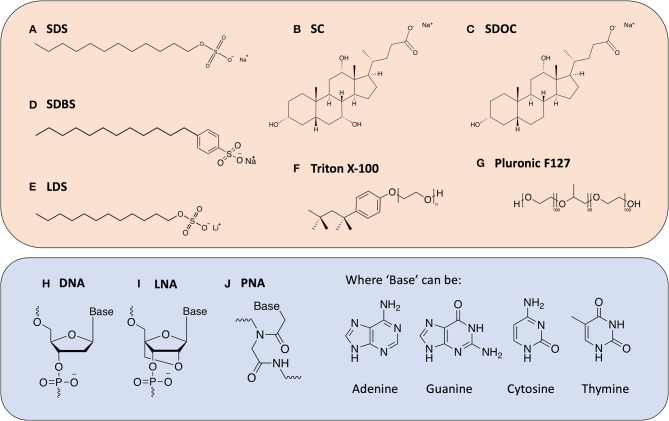
Chemical structures of the various dispersants discussed in this review. Surfactant molecules (top box) **(A)** Sodium Dodecylsulfate (SDS); **(B)** Sodium Cholate (SC); **(C)** Sodium Deoxycholate (SDOC); **(D)** Sodium Dodecylbenzenesulfonate (SDBS); **(E)** Lithium Dodecylsulfate (LDS); **(F)** Triton X-100; **(G)** Pluronic F127; and oligonucleotide-derived molecules (bottom box); **(H)** Deoxyribo Nucleic Acid (DNA); **(I)** Locked Nucleic Acid (LNA); **(J)** Peptide Nucleic Acid (PNA).

Since covalent functionalization of the nanotube surface is known to strongly affect, or even diminish, the nanotube fluorescence, non-covalent modifications are typically used for creating optical sensors. The most common approach for non-covalently separating SWCNT bundles is liquid-phase exfoliation and stabilization (Coleman, 2009). This approach typically involves using forced dispersion (with sonication, for example) in the presence of wrappings, such as surfactants, synthetic polymers, oligonucleotides, and proteins that can stabilize the suspended SWCNTs (Figure 2). In addition to improving the solubility of the SWCNTs, these wrappings can also impart secondary characteristics, such as enhanced bio-compatibility and improved molecular sensitivity, overcoming problems associated with the chemical inertness of raw SWCNTs (Saifuddin et al., 2013).

In this review, we present an overview of several key methods used for the non-covalent functionalization of SWCNTs. Beginning with surfactant-coated SWCNTs, we progress toward the use of biomolecules to suspend nanotubes, highlighting key advantages and disadvantages associated with each wrapping. Finally, we conclude with a consideration of new approaches aimed at overcoming some of the limitations of both surfactant- and biomolecule-suspended SWCNTs. These examples highlight emerging methods to selectively engineer improved SWCNT-based optical sensors in complex environments.

## 2. Surfactant-Coated SWCNTs

Surfactant-coated SWCNTs represent a standard comparative benchmark for nanotube suspensions, particularly with respect to achieving scalable wrapping procedures and the high quantum yields necessary for optical sensing. Historically, the first reported suspensions of individual SWCNTs were achieved using an aqueous surfactant, sodium dodecylsulfate (SDS) (O'Connell et al., 2001, 2002; Bachilo et al., 2002). The resulting isolation of the nanotubes from surrounding bundles greatly improved the optical resolution of the absorbance spectra. Additionally, the authors were able to characterize the direct band gap of individual semiconducting SWCNTs with fluorescence spectroscopy (Bachilo et al., 2002; O'Connell et al., 2002), which was first hypothesized in the early 1990s (Dresselhaus et al., 1992; Hamada et al., 1992; Saito et al., 1992) and previously detected using Raman and STEM (Wildoer et al., 1998; Kataura et al., 1999).

**Figure 3:**
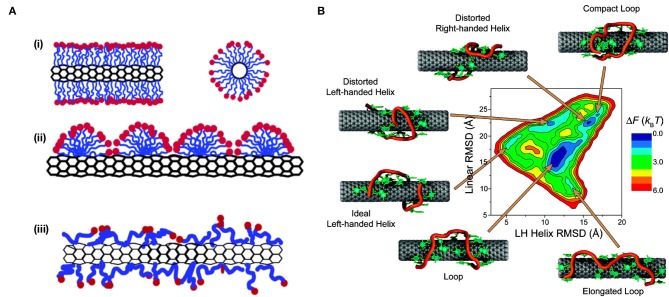
Conformations of non-selective wrappings on SWCNT surface. **(A)** A schematic representation of the various mechanisms by which surfactant molecules interact and disperse SWCNTs. **(i)** Cylindrical micelle encapsulation; **(ii)** Hemimicellar adsorption; and **(iii)** Random adsorption. Reprinted with permission from Yurekli et al. (2004). Copyright © 2004, American Chemical Society. **(B)** Free energy landscape of (GT)_7_-SWCNT hybrid at room temperature. Representative conformations for each local minimum are displayed. The sugar-phosphate backbone is depicted in orange, and bases are shown in green. Reprinted with permission from Johnson et al. (2009). Copyright © 2009, American Chemical Society.

To prevent re-bundling and obtain a thermodynamically stable suspension, the strong cohesion energy of the isolated tubes (~120 kT nm^−1^) must be compensated by either tube-solvent, or in the case of surfactant-suspended SWCNTs, tube-surfactant interactions (Angelikopoulos and Bock, 2012). However, SWCNT suspensions often exist in a kinetically meta-stable state. Kinetic stabilization does not fully overcome the cohesion energy of the tubes; instead the surfactant creates a repulsive force between the tubes that reduces the likelihood of forming tube-tube contacts, hence slowing aggregation (Angelikopoulos and Bock, 2012). Similar to the interactions in the kinetic stabilization of colloids (Tummala and Striolo, 2008, 2009; Angelikopoulos and Bock, 2012; Kato et al., 2012; Oh et al., 2013). Previous studies hypothesized that the individual nanotubes are encased in the hydrophobic interiors of the micelle. The hydrophilic part of the surfactant molecules is believed to face outwards, creating a cylindrical micelle and a repulsive force between the nanotubes that renders a thermodynamically meta-stable suspension (Figure 3) (Angelikopoulos and Bock, 2008, 2012).

The use of surfactants to suspend SWCNTs has since expanded to include other anionic, cationic, and non-ionic surfactants (Hirsch, 2002; Wenseleers et al., 2004; Crochet et al., 2007; Haggenmueller et al., 2008; Blanch et al., 2010; Bergler et al., 2016; Nonoguchi et al., 2018), such as sodium cholate (SC), sodium deoxycholate (SDOC), sodium dodecylbenzenesulfonate (SDBS), lithium dodecyl sulfate (LDS), Triton X-100, and pluronic F127. Depending on the surfactant, high-quality dispersions can be achieved with large populations of individualized nanotubes (Coleman, 2009) and SWCNT concentrations >1 mg/mL. However, different surfactants have been found to vary greatly in the degree of dispersion and stability of the resulting suspensions. This variation is, in part, attributed to the interactions between the surfactant and nanotube, which result in the formation of different structures with varying degrees of surface coverage (Matarredona et al., 2003). In addition to cylindrical micelle SWCNT encapsulation, as was proposed for the SDS-suspended SWCNTs, two additional configurations include (Angelikopoulos and Bock, 2012; Xin et al., 2013) (i) Langmuir-type (random molecular adsorption) layers and (ii) adsorbed spherical and hemispherical micelles (Islam et al., 2003; Vo et al., 2016; Vo and Papavassiliou, 2017) (Figure 3A). The latter, spherical and hemispherical micelle formation, is adopted only by strong amphiphiles that prefer higher curvature aggregates. This formation of hemimicellar aggregates on the surface of the SWCNTs typically involves adsorption of the surfactant onto the nanotube followed by the self-assembly of the molecules, which is enabled by diffusion along the nanotube surface (Vo et al., 2016). In contrast, the former, random adsorption of the surfactant on the SWCNT surface, is adopted by weakly amphiphilic molecules [such as flavin mononucleotides (FMN)] and bile acid surfactants (including SC and SDOC) where adsorption is competitive, i.e., follows a Langmuir isotherm (Angelikopoulos and Bock, 2010, 2012; Tummala et al., 2010; Bergler et al., 2016; Xu et al., 2017).

According to both experiment and simulation, the degree of exposed SWCNT surface coverage following adsorption of surfactant molecules under all three regimes is largely dependent on surfactant concentration (Matarredona et al., 2003). Indeed, Wang et al. (2004) have shown that for Triton-X, the optimal surfactant dispersion concentration is different from the critical micelle concentration (CMC) and results from competition between maximizing surfactant adsorption on the nanotube surface and micelle-mediated depletion interactions between adjacent SWCNT bundles. Moreover, previous reports have shown vast differences in the maximum relative solubility of SWCNT complexes using the same surfactant, with an apparent dependence on the processing method and conditions. For example, suspensions made with SDBS can have maximum SWCNT concentrations of 20 mg/mL (Islam et al., 2003) or no more than ~0.01 mg/mL (Moore et al., 2003), depending on the dispersion approach.

Another factor believed to impact the stability of surfactant-dispersed SWCNTs is the ζ-potential. When surfactant molecules adsorb onto the surface of SWCNTs, the surfactant counter-ion (commonly Na^+^ or Li^+^) is dissociated from the hydrophilic head group of the surfactant. These counter-ions are spatially separated from the tail group of the molecular ions, arranging in a diffuse cloud that acts as a multi-pole. As a result, surfactant-suspended nanotubes appear, from a distance, to carry an effective charge associated with this double layer, which is denoted as the ζ-potential (Coleman, 2009). This potential is equivalent to the electrostatic potential measured at the edge of the layer at the bound surfactant tail groups, and it acts as a repulsive interaction potential between neighboring SWCNTs. In a study by Sun et al. (2008), the dispersion quality of six surfactant molecules was tested. Each of the dispersion-quality metrics were found to scale well with the measured ζ-potential of the dispersion, with SDS suspending better than both SDBS and SC, corresponding to ζ-potential values of −70.0, −68.8, and −48.8 mV, respectively. These findings indicate that the dispersion quality of surfactant-SWCNTs may be controlled by the magnitude of the electrostatic repulsive forces between the coated SWCNTs (White et al., 2007; Sun et al., 2008), a property that can be tuned in order to improve the long-term stability of these solutions.

Given the dependence of maximum dispersion concentration and stability on surfactant type, we focus the remainder of our discussion on the four most commonly used surfactants for SWCNT suspension, SC, SDOC, SDS, and SDBS. These surfactants have been shown to achieve stable dispersions with suspension efficiencies above 40% (Haggenmueller et al., 2008). Despite the similar structures of SC and SDOC, which only differ by a hydroxy group, SDOC shows a marked increase in suspension yield (+17%). In addition to dispersion efficiency, the resolution of the optical absorption spectrum can be used to determine differences in the quality of SWCNT suspensions. Distinct absorption peaks are observed for both SC and SDOC, while SDS and SDBS show much broader peaks. In instances where SWCNTs are not effectively exfoliated, the van der Waals interactions between aggregated nanotubes result in broad, weak absorption peaks (Antonucci et al., 2017). This observation therefore suggests that SC and SDOC can generally yield more monodisperse SWCNTs under the studied preparation conditions. On the other hand, the broader peaks observed for SDS and SDBS indicate that these surfactants do not effectively de-bundle the nanotubes, resulting in a poorer dispersion quality despite the apparently high suspension yields.

In addition to their high dispersion efficiencies, these surfactants also benefit from a number of additional advantages. Compared to most biopolymers, these wrappings yield SWCNT suspensions that are relatively cheap and stable, and the preparation procedures are scalable enough to produce large volumes of monodisperse SWCNTs, which is an important consideration for the industrialization of nanotube sensors. Furthermore, surfactant-suspended SWCNTs typically exhibit much larger suspension (Coleman, 2009) and quantum yield values (Haggenmueller et al., 2008) compared to both protein- and DNA-suspended SWCNTs. The increased fluorescence intensity is attributed, in part, to the increased surface coverage of the surfactant on the SWCNT surface. The increased coverage results in higher levels of oxygen and water shielding, which has been shown to decrease nanotube fluorescence (Zheng et al., 2017), thereby leading to brighter SWCNT complexes. This increase in brightness is particularly important for biosensing applications, where penetration depth and sensor sensitivity have been linked to quantum yield (Yum et al., 2013; Bonis-O'Donnell et al., 2017, 2019; Beyene et al., 2018).

Toxicity is an additional metric when considering surfactant-suspended SWCNTs for *in vitro* and *in vivo* biosensing applications. Surfactants allow SWCNTs to disperse in water, the universal and biological solvent, permitting researchers to flexibly carry out a variety of environmental, biocompatibility, and safety analyses (Coleman, 2009). However, certain surfactants, such as SDS and SDBS, are known to be cytotoxic to cells even at concentrations as low as 0.05 mg ml^−1^ (Dong et al., 2008), and similar effects have been observed for nanotubes suspended with these surfactants (Dong et al., 2008, 2009). In studies performed by Dong et al. (2008) and Dong et al. (2009), neither the proliferation nor viability of the cells was affected by pristine SWCNTs in the absence of surfactant. Furthermore, conjugates of SWCNTs suspended with various concentrations of SC also showed no negative impact on cell viability and growth, and proliferation was comparable to that of untreated cells. The observed cytotoxicity of the nanotube-surfactant conjugates was therefore believed to be driven by the inherent cytotoxicity of the surfactant in the suspension (Dong et al., 2008, 2009). These studies illustrate the importance surfactant selection in overcoming challenges in toxicity. Although issues such as toxicity can be mitigated through appropriate selection of surfactant type and concentration, surfactant-suspended SWCNTs are limited for biosensing applications due to their lack of inherent selectivity. As a result, current efforts focus on the use of alternative wrappings to suspend SWCNTs, including biopolymers, such as single-stranded DNA (ssDNA) and proteins.

## 3. Biopolymer-Suspended SWCNTs

DNA is one of the most extensively studied wrappings for optical sensing applications based on Raman, fluorescence, and absorption spectroscopies (Zheng et al., 2003a; Heller et al., 2006; Enyashin et al., 2007; Zhang et al., 2011; Bansal et al., 2013; Kupis-Rozmysłowicz et al., 2016; Wu et al., 2018). The non-covalent functionalization of ssDNA is based on π-stacking of the aromatic nucleotide bases with the sp^2^-hybridized side-wall of carbon nanotubes (Zheng et al., 2003a). This arrangement exposes the negatively charged sugar-phosphate backbone of the DNA, which is hydrophilic and easily solvated, toward the water, enabling suspension of the DNA-SWCNT complexes in aqueous solutions (Zheng et al., 2003a). These favorable side-wall-DNA and DNA-water interactions yield suspensions that are facile and stable at room temperature for several months (Zheng et al., 2003a). Work carried out by Zheng et al. (2003a) showed that almost any ssDNA sequence could be used to successfully suspend SWCNTs in the presence of a denaturant and mild sonication. Although atomic force microscopy (AFM) measurements and simulations show DNA to helically self-assemble around the SWCNT (Zheng et al., 2003a,b), the final binding structure has been shown to be sequence-dependent, and short ssDNA strands may also assume other configurations on the nanotube surface (Zheng et al., 2003a; Johnson et al., 2008, 2009) (Figure 3B). The sparser surface coverage of the DNA compared to surfactants such as SC exposes a larger carbon surface to water, resulting in a decrease in the intensity and emission energy of the SWCNT fluorescence. For example, the (7,5) chirality undergoes a bathochromic shift of 17.6 meV (15.6 nm) when wrapped in ssDNA instead of SC due to the greater water accessibility of the DNA wrapping and the resulting increase in the local dielectric constant at the nanotube surface (Jeng et al., 2006; Li and Shi, 2014). Such changes in the local dielectric have been shown to yield an expected fluorescence shift in SWCNT emission peaks (Choi and Strano, 2007).

In addition to the facile suspension procedure and stable assembly, ssDNA benefits from additional features ideal for scale-up sensor design. DNA-wrapped SWCNT suspensions can be further concentrated to achieve dispersion yields as high as 4 mg ml^−1^ (Zheng et al., 2003a). Additionally, the nearly limitless variability in sequence length and composition, as well as the well-established chemistries available for DNA functionalization, make ssDNA an incredibly malleable polymer for tuning the characteristics of the suspended SWCNTs. For example, Zheng et al. modified DNA-SWCNTs at one end with biotin that was used for immobilization onto streptavidin-coated beads (Zheng et al., 2003a). This study demonstrates one of many biochemical approaches for controlling DNA-SWCNT behavior by specifically engineering DNA-SWCNT complexes. Furthermore, both sequence length and base composition has been shown to impact the interaction potential of ssDNA with the surface of SWCNTs (Zheng et al., 2003a; Safaee et al., 2019), which has also recently been shown to vary with SWCNT chirality (Jena et al., 2017; Safaee et al., 2019).

The ability of DNA to form chirality-specific interactions has been exploited for a variety of applications, most notably, chirality separation. Chirality separation is key for multi-modal sensing applications where each chirality selectively responds to a distinct analyte in a solution mixture. Following separation, the individual chiralities can each be functionalized with a wrapping that selectively responds to a particular analyte of interest, and the analyte is detected by monitoring the corresponding wavelength. Many separation mechanisms have been devised (Chattopadhyay et al., 2003; Krupke et al., 2003, 2004; Zheng et al., 2003a,b; Heller et al., 2004; Strano et al., 2004; Huang et al., 2005; Lustig et al., 2005; Arnold et al., 2006; Peng et al., 2006; Zheng and Semke, 2007; Tu and Zheng, 2008; Tu et al., 2009; Zhang et al., 2015) with varying degrees of success; however, a facile approach for scalable, complete and total fractionation into all the single chiralities remains elusive. Aqueous two-phase polymer (ATP) separation (Khripin et al., 2013; Ao et al., 2014, 2016; Ao and Zheng, 2015; Subbaiyan et al., 2015) has emerged at the forefront of methods currently employed in chirality separation. Briefly, an ATP system consists of two separate, but permeable, water phases of slightly different compositions that is created by polymer phase separation (Ao et al., 2016). Studies have shown that the partitioning of DNA-SWCNT complexes has a strong dependence on both the DNA sequence and SWCNT structure (i.e., chirality) (Ao et al., 2014). Moreover, this partitioning can be modulated by changing the polymer compositions of the two phases in order to rescale the hydration energies. For example, the addition of dextran (DX) to a poly-(ethylene glycol)/polyacrylamide (PEG/PAM) system pulls down DNA-SWCNTs from the top to the bottom phase (due to increased hydrophilicity) while the addition of poly(vinylpyrrolidone) (PVP) has the opposite effect. The effectiveness of this method was demonstrated in work carried out by Ao et al. (Khripin et al., 2013; Ao et al., 2014, 2016; Ao and Zheng, 2015), where over 300 DNA sequences were screened using ATP separation techniques, resulting in the isolation of 23 different chiralities.

**Figure 4:**
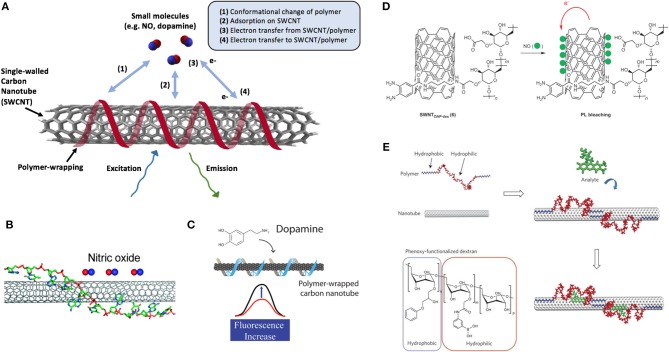
Non-specific interactions of polymer wrappings on SWCNT surface. **(A)** Common mechanisms of interaction between small molecules and polymer-wrapped SWCNTs. **(B)** The optical response of (AT)_15_ DNA oligonucleotide-wrapped SWCNT [(AT)_15_-SWCNT] upon exposure to NO. A side schematic view of one of the several binding structures of (AT)_15_-SCWNT simulated from a HyperChem simulation package. Bases of the DNA stack on the sidewall of the SWCNT, and the sugar-phosphate backbone extends away from the surface. Adapted with permission from Zhang et al. (2011). Copyright © 2011, American Chemical Society. **(C)** Schematic of the fluorescent turn-on sensor for dopamine. Adapted with permission from Kruss et al. (2014). Copyright © 2006, American Chemical Society. **(D)** Schematic illustration for NO detection using SWCNT-polymer hybrids, highlighting the mechanism for near-IR fluorescence bleaching by NO with SWCNT-DAP-dextran complexes. PL = photoluminescence. Reprinted with permission from Kim et al. (2009). Copyright © 2009, Nature Publishing Group. **(E)** A synthetic polymer with an alternating hydrophobic-and-hydrophilic sequence adopting a specific conformation when adsorbed to the nanotube. The polymer is pinned in place to create a favored recognition site for the molecules of interest, leading to either a wavelength or intensity change in SWCNT fluorescence. Adapted with permission from Zhang et al. (2013). Copyright © 2013, Nature Publishing Group.

Aside from their chirality specificity and selectivity, different DNA lengths and sequences have also shown preferences toward molecular recognition with certain analytes (Figure 4). Small nucleotide and microRNA sequences are promising biomarkers for a variety of pathologies, including cancer (Harvey et al., 2017). However, current methods of detection are complex and time-consuming, leading to difficulties in their implementation for point-of-care diagnostics. An advantage of DNA-SWCNT optical sensors is the ability to engineer selectivity toward target oligonucleotides by taking advantage of DNA's natural preference for specific complementary base pairing. Many studies have demonstrated the use of DNA-SWCNTs to quantitatively detect a range of both microRNA and DNA sequences (Jeng et al., 2006, 2010; Bansal et al., 2013; Harvey et al., 2017). Work carried out by Jeng et al. and Harvey et al. have shown that these fluorescence-based sensors are even capable of detecting single nucleotide polymorphisms (SNPs) (Jeng et al., 2010) and can be multiplexed to detect several sequences simultaneously (Harvey et al., 2017). In the study by Jeng et al., the addition of complementary DNA is believed to increase the surface coverage of the SWCNT upon hybridization, resulting in a decrease in the effective dielectric constant of the surrounding SWCNT environment and a shifting of the SWCNT fluorescence peak. Similarly, Harvey et al. propose an underlying mechanism based on changes in dielectric constant and electrostatic charge, which can modulate SWCNT emission wavelengths upon hybridization. The fluorescence shifting observed in both studies in response to complementary hybridization is particularly advantageous for detecting diseases, such as heart and kidney disease, as well as various cancers, which can be associated with different combinations of specific microRNA sequences (Etheridge et al., 2011; Hayes et al., 2014; Mishra, 2014; Bertoli et al., 2015; Wang et al., 2016; Hamam et al., 2017).

In addition to detecting hybridization, DNA-SWCNTs can also be engineered to detect a variety of other molecules, including neurotransmitters, sugars, and peptides (Xu et al., 2007; Kruss et al., 2017; Landry et al., 2017; Bisker et al., 2018), though the underlying mechanism of these sensors remains an ongoing area of research (Bisker et al., 2015; Ulissi et al., 2015). While certain DNA-SWCNT sensors are based on oligonucleotides that act as molecular sieves, like the (AT)_15_-SWCNT sensors designed to detect NO (Zhang et al., 2011), an alternative approach is based on displacement or conformational changes of the DNA wrapping (Heller et al., 2006; Landry et al., 2014, 2017; Salem et al., 2017; Beyene et al., 2018; Gillen et al., 2018). Early studies in this area screened libraries of molecules of interest against SWCNTs suspended using several different DNA sequences by monitoring the changes in the fluorescence emission of these sensors upon addition of the analyte. Through this approach, researchers were able to identify particular sequences with an enhanced affinity to certain chemicals, such as (AT)_15_ toward nitric oxide (NO) (Zhang et al., 2011) (Figure 4B) and (GT)_15_ toward catecholamines (Zhang et al., 2011; Kruss et al., 2017; Mann et al., 2017) (Figure 4C). Further studies have demonstrated that DNA length can also be used to tune the fluorescence properties of DNA-SWCNT hybrids, offering a new approach to controlling the behavior of these sensors (Jena et al., 2017; Beyene et al., 2018).

Recent studies carried out by Landry et al. and Lee et al. have shown that DNA aptamers on SWCNT scaffolds can be used to detect certain biologically relevant proteins (Landry et al., 2017; Lee et al., 2018). This label-free fluorescence detection offers many advantages over conventional immunological analytical methods, such as enzyme-linked immunosorbent assays (ELISA) or mass spectroscopy, which lack temporal resolution for real-time quantification of protein levels. Furthermore, this method obviates cumbersome purification and labeling steps typically required by more classical approaches. Both RAP1 and HIV-1 (Landry et al., 2017) and platelet-derived growth factor (PDGF) (Lee et al., 2018) were successfully detected using DNA aptamer-SWCNT complexes. Moreover, the RAP1 and HIV-1 sensors were also reported to selectively respond to their target proteins in molecularly complex environments, such as crude, unpurified cell lysates (Landry et al., 2017).

Although DNA-SWCNTs have shown improved selectivity toward small molecules compared to surfactant-SWCNTs (Zhang et al., 2013), they still lag behind their protein- and peptide-based counterparts, which offer exceptional molecular recognition. Proteins are capable of not only differentiating between molecularly similar targets, but also different chiralities of the same molecule. For example, whereas proteins, such as glucose oxidase (GOX) selectively interact with D-glucose (Zubkovs et al., 2017), sensors based on the (GT)_15_ DNA wrapping interact with a family of catecholamines (Kruss et al., 2014, 2017; Mann et al., 2017). Although glucose sensors based on DNA-SWCNTs have also been developed, these sensors ultimately require the addition of GOX for specificity due to the structural similarities of competing sugar molecules. Furthermore, the underlying sensing mechanisms for protein-based sensors are often more clearly identifiable. Their sensing mechanisms are quite diverse, varying from protein charge-transfer (Barone and Strano, 2006; Zubkovs et al., 2017) to exciton quenching due to protein conformational changes (Yoon et al., 2011), both of which have been shown to alter the SWCNT fluorescence intensity.

Protein-based wrappings, however, suffer from their own disadvantages; a lack of precise control during the protein immobilization process, for example, can result in unfavorable orientations that limit access to the active site (Mohamad et al., 2015). Similarly, structural rearrangements may occur that inhibit, or in some cases destroy, the bioactivity of these molecules (Saifuddin et al., 2013; Antonucci et al., 2017). In addition, protein-based wrappings exhibit limited dispersion efficiency, which has been shown to depend on the protein and is generally less efficient than DNA- and surfactant-based suspensions. Several methods of functionalization have been proposed (Huang et al., 2002; Jiang et al., 2004; Gao and Kyratzis, 2008; Saifuddin et al., 2013; Antonucci et al., 2017) and used to create sensors based on Luciferase-suspended SWCNTs (Kim et al., 2010), AnnexinV-suspended SWCNTs (Neves et al., 2013), and anti-uPA-suspended SWCNTs (Williams et al., 2018), for example. However, these sensors require an intermediate linker or wrapping for stability, as opposed to the non-specific adsorption possible with GOX. In fact, other protein-based glucose sensors, such as those based on glucose-binding protein (Yoon et al., 2011; Yum et al., 2013), typically require more complex conjugative chemistries compared to GOX, highlighting the importance of understanding the underlying protein mechanism when determining the most appropriate method for functionalization. Although these intermediate wrappings can improve solubilization and help maintain protein structure and function on the SWCNT surface, more complex functionalization procedures with multiple conjugation steps could limit the scalability of the sensors.

Irrespective of the improved selectivity offered by DNA and especially protein-suspended SWCNTs, these sensors suffer from relatively low quantum yields compared to surfactant-suspended nanotubes (Haggenmueller et al., 2008). The low intensities restrict the depth at which these biosensors can be implanted for use *in vivo* and *in vitro*. Moreover, studies have shown that both protein- and DNA-SWCNTs are sensitive to local variations in pH (Nepal and Geckeler, 2006; Antonucci et al., 2017) and ionic strength (Heller et al., 2006; Holt et al., 2012; Salem et al., 2017; Gillen et al., 2018). The latter poses additional challenges for biosensing applications, as these ions are often involved in biological signaling pathways (such as Ca^2+^ in dopamine regulation). Therefore, fluctuations in local concentrations throughout the day would compromise the sensing capabilities of the DNA-SWCNT complexes.

## 4. Polymer Engineering of SWCNT Sensor Specificity

The tradeoffs between surfactant-suspended SWCNTs and biopolymer-suspended SWCNTs have encouraged researchers to seek an alternative means of detection based on synthetic or bioengineered polymers. *Xeno* nucleic acids (XNAs), for example, have recently been engineered to improve the sensing capabilities of DNA-SWCNTs in ionically complex systems (Gillen et al., 2018). XNAs are synthetic alternatives to naturally occurring DNA and RNA that typically benefit from greater resistivity against nuclease degradation. Due to their modularity, nucleic acids can be readily adjusted using a variety of chemical modifications (Pinheiro and Holliger, 2012; Pinheiro et al., 2013; Ghosh and Chakrabarti, 2016; Ma et al., 2016), and XNAs can contain modifications to either the nucleobase, phosphate, or sugar in an otherwise native oligonucleotide sequence (Pinheiro et al., 2013; Pinheiro and Holliger, 2014; Anosova et al., 2016). Although XNAs were initially developed to emulate the DNA replication processes found in nature, these synthetic oligomers were quickly realized for their advantages in *in vivo* stability and specificity (Wang et al., 2005; Pinheiro and Holliger, 2014; Taylor et al., 2014; Ma et al., 2016). Larger base modifications can result in altered physico-chemical properties, such as a tendency to adopt non-standard helical conformations, but certain chemical modifications to the N7 (in purines) or C5 (in pyrimidines), sites that extend into the major DNA groove, can be reasonably tolerated without significant steric impact (Pinheiro and Holliger, 2012). Backbone modifications can also alter the physico-chemical properties of oligonucleotides. One example is peptide nucleic acid (PNA), where the sugar phosphate backbone is substituted with aminoethylgylcine. This substitution results in a charge-neutral polymer that is capable of strong canonical base pairing. The type and extent of the modification depends on the intended application. For example, locked nucleic acid (LNA) can greatly improve the stability of SWCNT sensors in the presence of high ionic concentrations (Gillen et al., 2018). Previous studies showed that salt cations can alter the DNA conformation on the nanotube surface, changing the emission wavelengths (Heller et al., 2006; Salem et al., 2017; Gillen et al., 2018). Since the added methyl bridge in the backbone of LNA increases the rigidity of the polymer, LNA exhibits increased conformation stability in the presence of fluctuating salt concentrations. By modifying 25% of the sequence with a “locked” base, bioengineered sensors based on LNA have been shown to withstand over two orders of magnitude higher salt concentrations without any perturbations in fluorescence. These complexes offer a strong promise for use in ionically complex media, such as blood or urine, without compromising the biocompatibility or nearly inexhaustible sequence space offered by oligonucleotide wrappings. The added chemical modifications also carry untapped potential for further narrowing selectivity through bio-conjugative chemistries that are specific to functional groups in the desired target.

Similarly, recent work by Chio et al. has employed the use of peptoids, N-substituted glycine polymers, to serve as protein molecular recognition elements for SWCNT-based sensors (Chio et al., 2019). These peptoids draw inspiration from biological peptides, with the benefit of greater resistivity against protease degradation (Anosova et al., 2016). The tunability of these sequence-defined synthetic polymers enables greater chemical diversity by providing a larger monomer space of primary amines (Sun and Zuckermann, 2013). Although the stability of the peptoid wrapping on the nanotube surface was shown to vary depending on composition, length, charge and polarity, Chio et al. demonstrated that these sensors could be used to engineer a selective sensor for the fluorescence detection of the lectin protein, wheat germ agglutinin (WGA) (Chio et al., 2019).

In addition to peptoids and oligonucleotide derivatives, purely synthetic heteropolymers have also been used to augment sensor properties. One such platform uses Corona Phase Molecular Recognition (CoPhMoRe) and relies on SWCNT-adsorbed heteropolymers to template preferential recognition sites for target analytes. The final structures adopted by the polymer on the surface control the selectivity of the sensor toward a target. Though the mechanism for modulating SWCNT fluorescence in response to binding is likely analyte- and polymer-specific, its precise characterization remains an area of active research (Bisker et al., 2015; Ulissi et al., 2015). Typically, the heteropolymers employed contain both hydrophobic and hydrophilic segments. The former interacts with the SWCNT surface, while the latter extends into solution to suspend the complex in aqueous solutions. CoPhMoRe-based sensors have been developed to detect neurotransmitters (Kruss et al., 2013; Zhang et al., 2013; Landry et al., 2014), vitamins (Zhang et al., 2013), and steroids (Zhang et al., 2013), as well as small molecules, such as NO and H_2_O_2_ (Kim et al., 2009; Iverson et al., 2013; Giraldo et al., 2015) (Figure 4).

Furthering the development of these sensors, Bisker et al. (2016) extended the capabilities of CoPhMoRe sensors to detect larger macromolecules, such as proteins. A variant of a CoPhMoRe screening approach was used to identify polymeric wrappings that could be used to create synthetic, non-biological antibody analogs capable of recognizing biological macromolecules. This approach yielded a selective sensor for fibrinogen based on dipalmitoyl-phosphatidylethanolamine (DPPE)-PEG (5 kDa)-suspended SWCNTs. This sensor was capable of detecting fibrinogen in a competitive binding assay in the presence of albumin, which can passivate the sensor by binding to non-specific binding sites (Bisker et al., 2016). This observation suggests that CoPhMoRe is more likely due to a combination of factors related to both the specific corona phase formed by the polymer-SWCNT complex and the unique elongated conformation of the fibrinogen protein, rather than sensing mechanisms based on aggregation, molecular weight, or protein hydrophobicity.

## 5. Conclusions and Future Perspective

Since the first reported aqueous suspension of individual SWCNTs with surfactant (O'Connell et al., 2001, 2002; Bachilo et al., 2002), SWCNTs have been suspended using a variety of natural and synthetic wrappings. Polymer wrappings in particular have served the dual purpose of both solubilizing SWCNTs and regulating the selectivity of SWCNTs toward specific analytes in biological media. As a result, polymers such as DNA have become standard wrappings for optical SWCNT-based biosensing, and recent efforts have focused on modifying these polymers to improve the quantum yield, stability, scalability, and selectivity of these sensors. However, with the exception of protein-based wrappings and complementary DNA-strand hybridization, the nature of the selectivity of polymer wrappings toward specific analytes remains unclear. As a result, most DNA and synthetic polymer-based SWCNT sensors are empirically engineered through random library screening and selection. These techniques evaluate the responsivity of several different polymer-wrapped SWCNTs against a variety of analytes, and the polymer-analyte combinations that yield relatively strong fluorescence responses are used to identify suitable polymer wrappings for SWCNT-based sensing (Zhang et al., 2011, 2013). Though this approach has been quite successful in identifying wrappings that can trigger a fluorescence response toward particular analytes, the sensors often show compromised selectivity. Moreover, the polymer wrappings also yield sensors with lower stability and brightness compared to surfactant wrappings.

Studies for new SWCNT optical sensors thus far screen, at most, tens of polymers at a given time (Zhang et al., 2011, 2013), meaning they have only explored a small fraction of the near-infinite polymer sequence space. One approach to overcoming the current limitations of SWCNT sensors is to screen larger polymer libraries in order to increase the chances of identifying a polymer-analyte combination with more favorable sensing properties. An alternative approach to addressing this challenge is to implement more guided techniques, such as directed evolution (Arnold, 1997). Directed evolution uses an iterative approach to improving the properties of materials that lack a defined structure-function relationship. Though the technique is conventionally used to engineer proteins, it was recently applied to engineer DNA wrappings that were shown to improve the quantum yield of an optical SWCNT-based sensor (Lambert et al., 2019). Combined with computational methods, such guided approaches can be used to identify trends between polymer sequence and sensor properties, with the goal of ultimately understanding the underlying mechanism for selectivity and designing molecular probes in a rational and predictive manner.

## Author Contributions

AG and AB contributed to the research and writing of this manuscript. Both authors have agreed on the final version of the manuscript submitted.

### Conflict of Interest Statement

The authors declare that the research was conducted in the absence of any commercial or financial relationships that could be construed as a potential conflict of interest.
